# The Ultra fit community mask—Toward maximal respiratory protection via personalized face fit

**DOI:** 10.1371/journal.pone.0281050

**Published:** 2023-03-15

**Authors:** Chulho Hyun, Mark M. Jensen, Kisuk Yang, James C. Weaver, Xiaohong Wang, Yoshimasa Kudo, Steven J. Gordon, Anthony E. Samir, Jeffrey M. Karp

**Affiliations:** 1 Katharos Labs LLC., Boston, Massachusetts, United States of America; 2 Harvard Medical School, Boston, Massachusetts, United States of America; 3 Department of Anesthesiology, Perioperative and Pain Medicine, Center for Nanomedicine, Brigham and Women’s Hospital, Boston, Massachusetts, United States of America; 4 Harvard–MIT Division of Health Sciences and Technology, Cambridge, Massachusetts, United States of America; 5 Proteomics Platform, Broad Institute, Cambridge, Massachusetts, United States of America; 6 Division of Bioengineering, Incheon National University, Incheon, Republic of Korea; 7 Research Center for Bio Materials & Process Development, Incheon National University, Incheon, Republic of Korea; 8 John A. Paulson School of Engineering and Applied Sciences, Harvard University, Cambridge, Massachusetts, United States of America; 9 Department of Radiology, Center for Ultrasound Research & Translation, Massachusetts General Hospital, Harvard Medical School, Boston, Massachusetts, United States of America; 10 Harvard Stem Cell Institute, Cambridge, Massachusetts, United States of America; National Textile University, PAKISTAN

## Abstract

Effective masking policies to prevent the spread of airborne infections depend on public access to masks with high filtration efficacy. However, poor face-fit is almost universally present in pleated multilayer disposable face masks, severely limiting both individual and community respiratory protection. We developed a set of simple mask modifications to mass-manufactured disposable masks, the most common type of mask used by the public, that dramatically improves both their personalized fit and performance in a low-cost and scalable manner. These modifications comprise a user-moldable full mask periphery wire, integrated earloop tension adjusters, and an inner flange to trap respiratory droplets. We demonstrate that these simple design changes improve quantitative fit factor by 320%, triples the level of protection against aerosolized droplets, and approaches the model efficacy of N95 respirators in preventing the community spread of COVID-19, for an estimated additional cost of less than 5 cents per mask with automated production.

## Introduction

The COVID-19 pandemic caused by the severe acute respiratory syndrome coronavirus (SARS-CoV-2) remains an unprecedented threat to global public health. SARS-CoV-2 is known to spread via respiratory droplets and aerosols asymptomatically and pre-symptomatically [1]. In the absence of cure or vaccination, numerous non-pharmaceutical public health interventions such as travel restrictions, mass screening, quarantine centers, and contact tracing have been implemented to control the pandemic [2]. Even after successful vaccination, masks can prevent potential asymptomatic spread, and provide protection for immunocompromised individuals and in communities where vaccination has been limited [3].

As a low-risk, precautionary control method [4], universal face masking has shown effectiveness in reducing disease spread [5]. However, the performance of most cloth and disposable 3-ply, 3-pleat disposable masks (3PM) is degraded by poor face fit, resulting in leakage of aerosols at gaps between the mask and a wearer’s face. This leakage reduces the quantifiable ‘fit factor’, a metric of mask fit performance. Conventional 3PM have fit factors (FFs) of only 2.6–4.4 [6, 7], equivalent to c. 20–40% leakage. A further limitation of conventional disposable masks occurs during coughing and sneezing, which elevate the mask and enlarge peripheral gaps.

N95 respirators fit tightly and provide protection from airborne pathogens but were designed for short-term use in high-risk environments, not in community settings. N95 respirators require fit testing and are uncomfortable to wear for extended periods, which leads to poor adherence, frequent mask removal, and even personal injury [8–10]. Other barrier face coverings (e.g. cloth masks or gaiters) offer less protection but are better tolerated by users due to comfort and ease of use [11]. Due to large variability in the performance of commercially available consumer masks, the American Society for Testing Materials (ASTM) and National Institute for Occupational Safety and Health (NIOSH) released new standards of mask to provide better community and workplace protection against the spread of airborne contagion [12, 13]. A central feature of these new standards is their requirements for leakage (mask fit), filtration, and breathability.

Poor mask fit is due to 1) mismatch between mask and face shape, and 2) looseness of the mask, usually due to inability to adjust mask tightness. A variety of secondary devices (e.g. mask fitters, extender straps) and techniques (e.g. knotting, tucking, double masking) have been developed to help address these limitations. However, these technologies have not been broadly adopted owing to reductions in comfort and convenience [14, 15]. A simple, well-tolerated, and efficacious face covering technology would address this unmet need and likely promote broader adoption and improve user experience and compliance.

We developed a novel Ultra Fit Mask (UFM) by adding three innovations to the conventional 3PM design: (i) a peripheral moldable element, (ii) an integrated ear loop tension adjustment, and (iii) an inner ‘cough-trapping’ flap layer designed to trap exhaled aerosols. Each design element was specifically considered for its ability to be incorporated into current automated mass manufacturing systems with minimal modification and cost. Since UFM uses existing filter materials and the 3-ply, 3-pleat structure, the filtration mechanism is unaltered. By incorporating easy-to-manufacture features directly into the designs of mass-produced disposable masks, we enable rapid implementation and maximize the potential impact of these design innovations.

## Materials and methods

### Ultra fit mask fabrication

Materials used in the UFM are readily available and inexpensive. For the base mask, a 3PM with 3 pleats was selected (SupplyAID, SnowJoe LLC, Hoboken, NJ) due to its availability. This 3PM is compliant with GB/T32610-2016. However, any rectangularly-shaped pleated face mask and medical-use surgical mask can be used as a base mask. As the supply of face mask improved and the ASTM F3502 standard emerged, a base mask compliant to NIOSH filtration test was used. Also, the dimensions of base mask were carefully determined by analyzing non-medical 3PM. A base mask with 175 x 95mm (pleated, as is), 175 x 160mm (unpleated), ear loop string lengths of 180-190mm was used for a broader population. In addition, based on surveying spring constants of elastic ear loop strings from 9 mask manufacturers, we used strings with less than 12 N/m at 50 grams. Strings with >12 N/m cause discomfort and pain due to more pressure exerted on the back of the ears. Four segments of Zinc-galvanized steel wires with a diameter of 0.023” (8872K16, McMaster-Carr, Elmhurst, IL) were incorporated on the four edges on the rear side of the base mask, which were then taped over with medical adhesive tape for soft skin contact (MedFix^™^, Medline, Northfield, IL). Empiric experimentation across a range of different wire diameters demonstrated the best performance with 0.50mm to 0.58mm bare wire, where experimentation was to deform a Ultra fit mask by placing it on the experimenter’s face and skin. The material of the inner flap layer was spunbond polypropylene nonwoven fabric with a weight of 30–40 grams per sq. meter (GSM), the identical material as the inner mask layer, which is a low-cost, non-hazardous material for skin contact. A 30–40 GSM fabric was chosen because of its reliable ultrasonic weldability. We observed sonic welding with a lower GSM fabric resulted in weak joining. Custom-designed ear loop adjusting components were cut from a stock polypropylene sheet (Formex-GK17, ITW, Carol Stream, IL) and bonded with hot-melt glue on the upper corners on the front side of the base mask. The bulk cost of materials is estimated to be less than $0.05 per mask. At the time of writing, we demonstrated that these modifications can also be performed with a single ultrasonic welding operation, eliminating the need for adhesives or tapes.

### Visualization of face seal leakage

For visualization of exhaled vapor, a custom laser particle scattering setup was devised. An e-cigarette was used to produce vapor particles with a mean particle size ranging from 174 to 236 nm [16]. Before donning a mask, the user inhaled vapor, donned the mask, and then exhaled. Particles were assessed using light scattered from a green laser pointer (Laser 303, λ_c_ = 532nm, P_max_ <5mW) equipped with a cylindrical lens (LK1395L1, Thorlabs, Inc., Newton, NJ) to create a linear beam. The beam illuminated sagittally toward the subject and coronally toward the chin. The exhalation event was recorded on an Apple iPhone X mounted on a tripod. For relative scattering analysis, the green channel of video files was analyzed over time using MATLAB. Relative scattering was calculated as a sum of pixel values divided by the segmented area. Plots were then normalized to the theoretical maximum. Also, the participant in Fig 2 has given written informed consent (as outlined in PLOS consent form) to publish the case details.

For infrared thermal imaging, two cameras, T440 (Teledyne FLIR, Wilsonville, OR) and CompactPro (Seek Thermal, Santa Barbara, CA) were initially used. For convenience of image transfer, CompactPro was used for image acquisition. The room temperature was maintained around 27 °C. The thermal camera was positioned about 1m away from the subject such that the subject’s head fills >50% of the image. The subject, sitting on a chair, breathes through the nose at rest for at least 2 minutes. For presentation purpose, images were cropped around the subject’s head. For plotting thermal variation over time, 5-by-5 pixel regions were segmented for the left and the right infraorbital regions and a 20-by-20-pixel region was segmented for the center of the mask (Fig 3A). Mean values of the segmented regions were plotted over time. Thermal video data was acquired at 8.6 frames/second.

An aerosol dye challenge was used to evaluate the protection provided by the mask to external aerosol exposure. The aerosol dye was generated 20 cm away from a head model, which was 3D printed using data obtained from the NIOSH Anthropometric Data and ISO Digital Headform [17], and was modified to outfit with a 2 cm tube connected to the mouth. To simulate inhalation and exhalation, a 2L resuscitator bag (Vodeson) was modified by closing the one-way valves on the back of the bag and removing the valve at the interface with the mouthpiece. The resuscitator bag was physically compressed and then allowed to expand in 3 second cycles over a 30 second test period to simulate breathing. Aerosol particles (27.94 ± 18.96 μm in diameter, 483,000 ± 102,000 particles per second, mean ±SD) of fluorescent dye (EC6 RECOLOUR Dye UV Green, XSPC) were generated from a home-use ultrasonic mesh nebulizer (Model MN-01A). Dye on the surface of the mannequin was imaged using a 365 nm ultraviolet light source (Model UVGL-25, UVP, LLC, Upland, CA) and a digital camera. The percent of the mannequin head’s surface coated in dye underneath the face mask was determined using image analysis in ImageJ 1.53E (National Institute of Health).

### Quantitative fit test

For a quantitative fit test, a commercial PortaCount^®^ Respirator Fit Tester 8038 was rented (Raeco Rents, LLC, Gurnee, IL) for the duration of experimentation. Automatic daily checks were performed before use. A standard protocol per 29 CFR 1910.134, which consisted of 8 exercises, was employed. After five exercises comprising different breathing styles (normal and heavy), head motions (side-to-side and up-and-down), and reading a provided passage, a wearer was asked to grimace to intentionally break a face seal, followed by bending over towards the toes before the final normal breathing was attempted. A fit factor of each exercise was automatically computed and displayed at the end of each exercise except for non-breathing ‘grimace’ exercise. An overall fit factor was automatically computed at the end of the whole exercise cycle. With N95 Companion mode enabled, PortaCount^®^ 8038 discriminates the contribution of leakage through the filter by use of electrostatic classifier and counts only particle sizes in the range of 25 nm to 60 nm. Therefore, the fit factors, as a measure of face seal leakage, are the ratio of ambient particle concentration outside the mask to that inside the mask for particle sizes between 25 nm and 60 nm. A conventional 3PM disposable mask without modifications and KF94 mask (AirQueen Nano, Soomlabs, Korea), a Korean standard rated to filter 94% of particles larger than 0.3 microns in size [18], were used as reference controls (S2 Fig). The 3PM used in our experiment was sold as SupplyAID by SnowJoe LLC, which also served as the base mask for UFM. A total of thirteen volunteers participated in the quantitative fit test.

### User validation

An online survey form was created and circulated for collecting user feedback. Questions asked in the survey were multiple-choice questions and short questions about what type of face mask a user wears, and the user’s assessment of the UFM. Results are available in the supplementary information.

### Modeling airborne infection risk mitigation

To evaluate how the design modification to the UFM would influence the risk of airborne pathogen spread we used the computational model developed by Peng et al. [19, 20]. Briefly, the model uses the concept of an enclosed box to estimate the amount of aerosol contamination taking into consideration: ventilation, volume of the space, and the amount of virus put into the air by infected individuals (factoring in mask leakage). The model then calculates an airborne risk parameter that considers the amount of virus in the air over the exposure period, the volume of air uninfected individuals takes in during breathing, and mask efficiency. The Wells-Riley model of infection is then used to calculate the probability of an individual becoming infected after exposure [21]. We derived the mask efficiency for the UFM based upon the results of the quantitative fit testing, filter testing, and the aerosol exposure test. For the cloth mask (mask efficiency 20%), excellent cloth (mask efficiency 50%), and N95 (mask efficiency 95%), reported mask efficiency values were used [22]. The mask efficiency values entered into the model generated in Peng et al. and the fraction of people with masks was varied systematically. The baseline settings for ventilation, volume, duration of stay, activity adjusted breathing rates, and infectious quanta for COVID-19 within the classroom and subway environments, were used. The number of individuals in the classroom and subway car were set to 20 and 35, respectively, and there was assumed to be one infective individual in each case. Data in the classroom was normalized against the risk of a secondary infection occurring in the absence of any masks over a 50-minute class period. As the duration of ride on a subway is highly variable, we calculated the absolute infection risk parameter, which is independent of the duration of the ride, at 0, 25, 50, 75, and 100% mask utilization.

### Statistical analysis

Data processing and statistical analysis were performed using a combination of MATLAB, ImageJ, and GraphPad Prism 9.0. All graphs and reported values represent mean ± standard deviation unless otherwise stated. A Shapiro-Wilks test was used to test if data were normally distributed before selecting parametric or nonparametric tests. Comparisons across multiple groups were performed on nonparametric data using Kruskal-Wallis for multiple groups and Mann-Whitney for paired tests. For parametric data, a one-way ANOVA was used. A Tukey post-hoc test was applied to correct for multiple comparisons and an adjusted p-value of < 0.05 was used as the threshold for significance. The specific test used is described in the caption of each figure.

### Ethics statement

Our quantitative fit test protocol was exempted by the Mass General Brigham Institutional Review Board (#2021P001871) as the protocol meets the criteria for exemption 45 CFR 46.101(b)(#). Also, the participant in Fig 2 has given written informed consent (as outlined in PLOS consent form) to publish the case details.

## Results

### Ultra fit mask—Improved mask design

**Fig 1:**
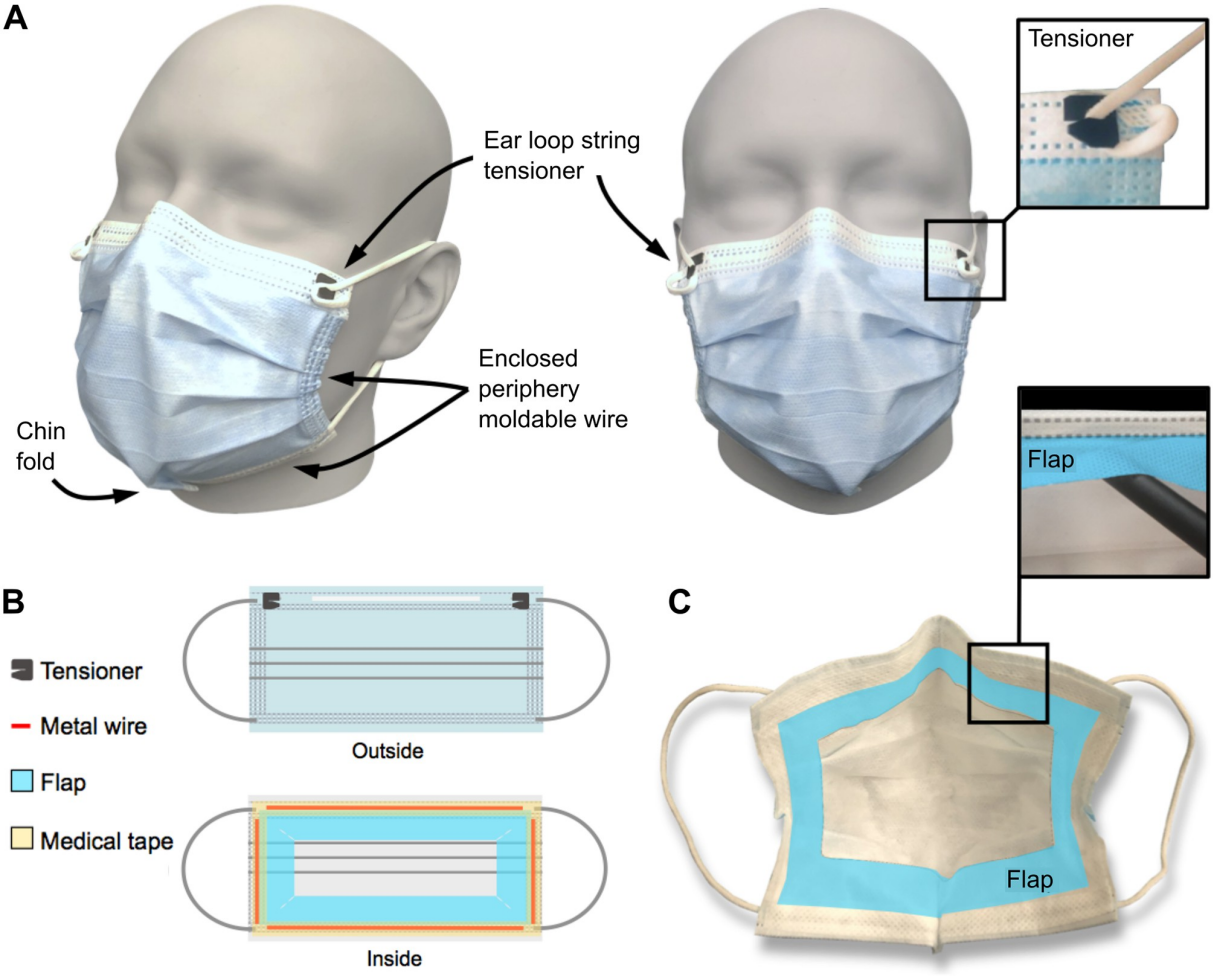
Ultra fit mask for reducing face seal leakage on the periphery of the mask body. (A) Ultra Fit Mask (UFM) fitted on a head form compliant to ISO 16976–2 Respiratory Protective Devices (medium size). (B) Exterior and interior schematics of prototype UFM. (C) Additional cough-trapping inner flap layer attached on the interior side is highlighted in blue.

To facilitate improved fit and seal gaps, we added a moldable wire element along the entire periphery of a conventional 3-ply disposable mask (3PM) (Fig 1A and 1B). With the moldable element, the mask gains a pliable structure that can conform to any face, with minimal gaps (S1 Movie). Compared to the 100 mm-long nose bridge wire found in non-medical 3PM, a longer and thicker nose wire (length > 120mm, diameter > 0.58mm) provides a better seal across the entire nasal-infraorbital region (bizygomatic breadth). The wires on the cheek and the chin edges of the mask provide a fitting experience similar to cup-shaped respirators and enable customized shaping of the mask edge. Pinching the side or chin wires allows to tighten the fit and further reduces gaps (S1 Fig). As such, these face-conformal adjustments improve fit, particularly for users with smaller faces.

To further promote a tight face seal, we added adjustable tension elements to the mask body. In conventional 3PM, elastic ear loop strings compress the mask onto the wearer’s face. The compressive force on a wearer’s face and the back of the ears is broadly a function of wearer head size or, precisely, the bitragion-subnasal arc (BSA) length, and the length and elasticity of the ear loop strings. Therefore, users with a longer BSA length suffer from irritation due to high pressure on the back of the ears while having a tight face seal, whereas other users with a shorter BSA length have a loose face seal without proper ear loop adjustment. To accommodate a broader population, in UFM, a longer ear loop strings were used, 180-190mm, which is 10-30mm longer than the ones found in 3PM. Also, elastic ear loops strings with a spring constant less than 12 N/m at a mass of 50g were used. Furthermore, a pair of tension adjustment components was integrated onto the base mask that cinches the elastic ear loop strings and allows length and tension adjustment by the users. Appropriate tensioning in combination with the face-conforming mask shape maximizes both comfort and face seal (Fig 1A).

To prevent the escape of droplets via the sides of the mask, we added an additional layer as a “cough trap” flap (Fig 1C). Coughing generates pressure that displaces the mask away from the face, reducing face seal exactly when it is needed most to capture rapidly ejected respiratory droplets. The incorporated inner flap serves to capture these droplets.

### Qualitative experiments of mask fit

#### Laser particle scattering of exhaled vapor

**Fig 2:**
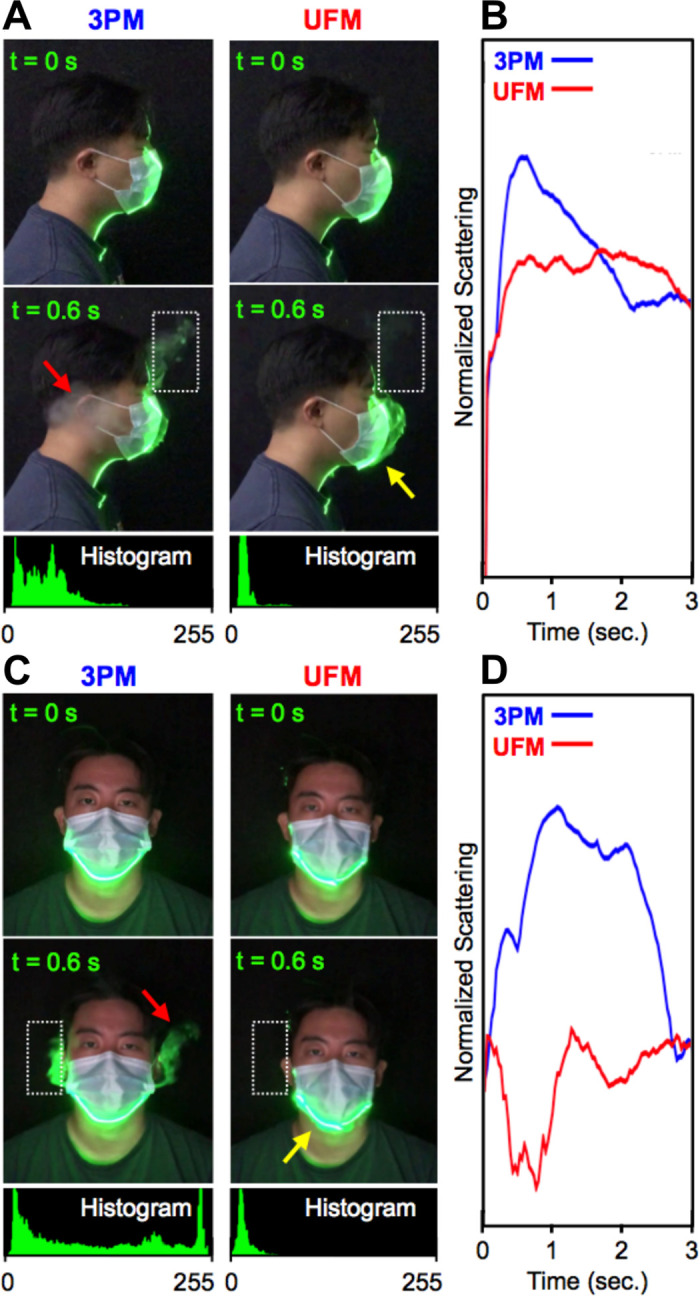
Evaluation of exhaled vapor using laser scattering. (A, C) Side-view and front-view exhaled vapor imaging and histograms of the segmented area (white dotted box). Red arrows indicate particles that escaped the mask, while yellow arrows indicate particles that passed through the mask filter material. (B, D) Normalized laser scattering of the segmented forehead and lateral regions over an exhalation period.

To evaluate the ability of the masks to reduce aerosol spread, we analyzed video recordings of vapor exhalation via laser light scattering as a qualitative assessment of mask fit. To image escaping vapor, video recordings were taken from the side and the front of a wearer (Fig 2A and 2C). Vapor escaping through open gaps for the 3PM was visible near the forehead, while vapor release through the side of the mask was clearly visible as a gray plume near the ears (Fig 2A, **red arrow**). Qualitative assessment of UFM vapor unequivocally demonstrated less vapor escape, with more vapor instead being forced through the mask filter material (Fig 2A, **yellow arrow**). These observations were confirmed by plotting relative laser scattering (RLS) of every frame of the acquired videos over time (Fig 2B and 2D). Leaked vapor from the UFM at the nasal-infraorbital side was nearly an order of magnitude less for the first 1.5 seconds than that with the 3PM (Fig 2B). After 1.5 seconds, a higher RLS with the UFM was observed, corresponding to scattering of floating vapor that penetrated the mask filter material earlier (S2 Movie). The UFM demonstrated negligible cheek leakage, with only a small leak plume arising from the chin region (Fig 2C, **yellow arrow**). With the UFM, the amount of laser scattering from around the cheeks due to leakage was reduced by ca. two orders of magnitude.

#### Infrared thermal imaging

**Fig 3:**
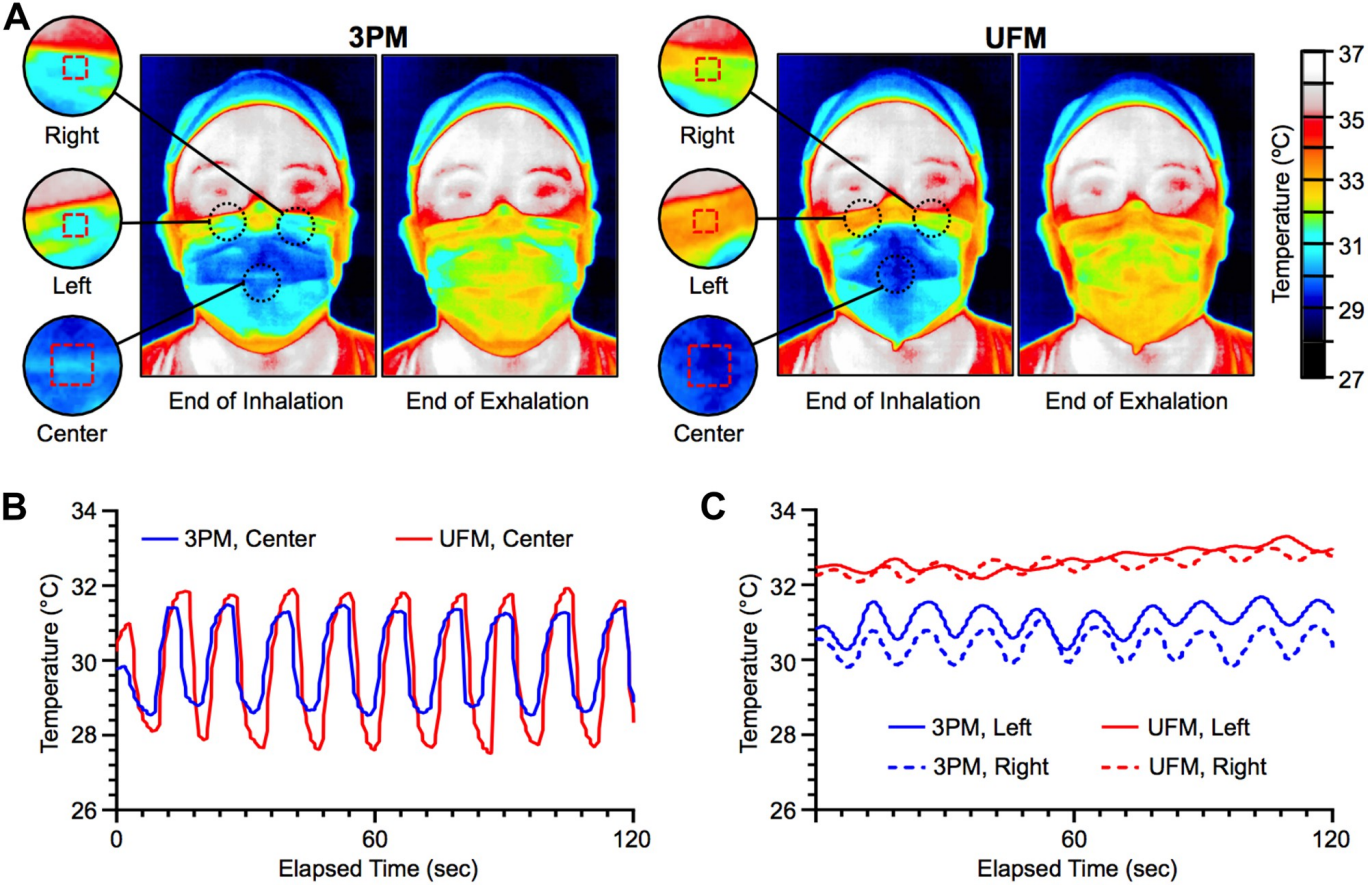
Infrared thermal imaging of mask surface over breathing cycles. (A) Infrared thermal snapshots at the end of inhalation and exhalation with 3-ply mask (3PM) and UFM were obtained from video recordings of breathing cycles. Three subregions at the left and right side of the nose (red dotted boxes, 5 x 5 pixels), and the center of the mask (red dotted box, 20 x 20 pixels) were selected for thermal variation analysis over multiple respiration cycles. Thermal variation of the (B) center subregion and the (C) left and right side of the nose over a 120-second breathing cycle.

To further assess leakage sites for both inhalation and exhalation, we used infrared thermal imaging during normal breathing in an air-conditioned room (S3 Movie). Synchronous thermal changes at the mask surface with breathing cycles were visible as relatively cool inhaled air and relatively warm exhaled breath pass through the mask filter material. Inward and outward leakage along the nose-infraorbital edge of the mask were visualized as zones of high thermal variation over time (Fig 3A). The ingress of cool air through the nasal-infraorbital edge of the mask at the end of inhalation and egress of warm breath at the end of exhalation indicated leakage for the 3PM. For the UFM, the nasal-infraorbital edge remained relatively warm during breathing cycles except for a small ingress on the right side of the wearer (Fig 3A). Over a 2-minute breathing cycle, the UFM showed larger temperature variation in the center of the mask consistent with cycling of cool inhaled air and warm exhaled air traversing the mask filter material (Fig 3B) Thermal variation analysis at the right and left sides of the nasal-infraorbital edge of both masks showed that a sinusoidal response in the UFM was less pronounced compared to 3PM. A higher offset temperature around the edge of the UFM was due to increased skin contact owing to the reduction in mask edge gaps (Fig 3C). Overall, thermal imaging demonstrated that the UFM directed air through the filter material and reduced ingress and egress air leaks around the mask edge.

#### Fluorescent aerosol dye challenge

**Fig 4:**
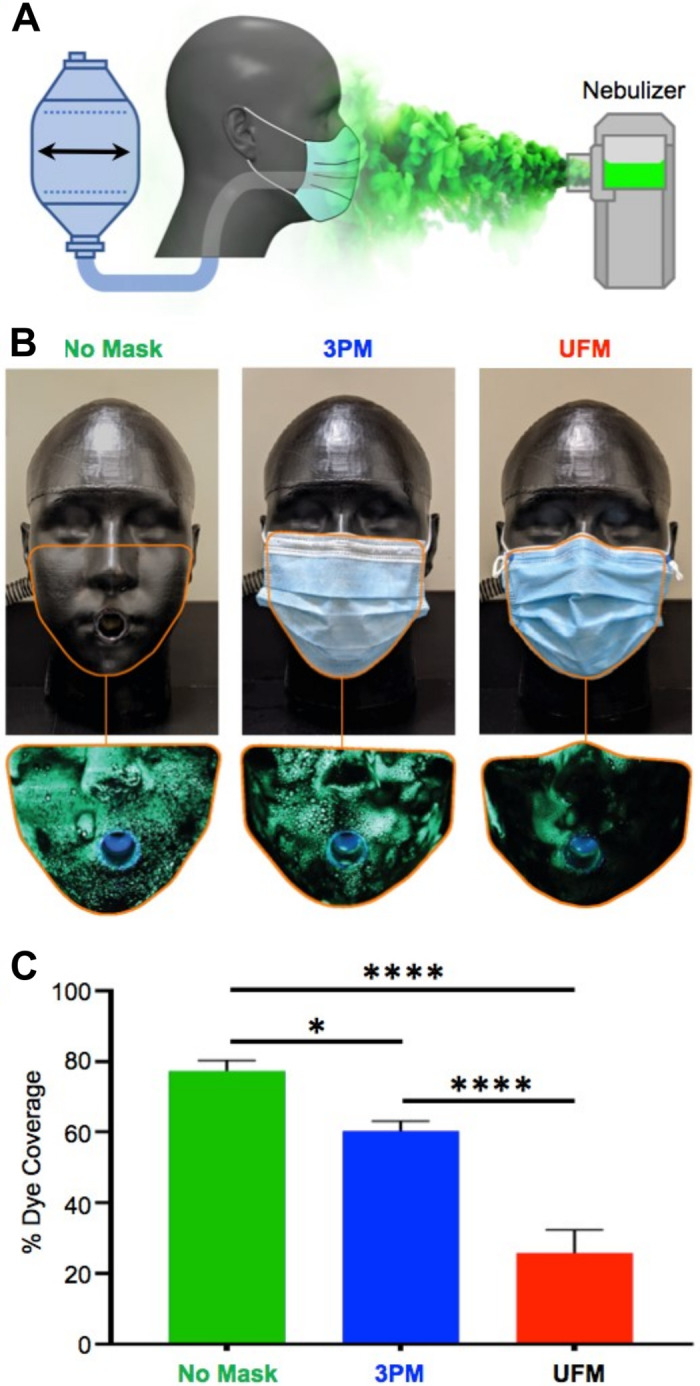
Aerosol dye examination of 3PM and UFM. (A) Schematic of the experimental setup where an aerosolized fluorescent dye was nebulized in the vicinity of the headform, with exhalation and inhalation breathing simulated by a modified resuscitator. (B) Fluorescent droplets deposited on a NIOSH medium headform with no mask, 3PM, and UFM. For scale, each image measures ca. 18 cm wide. A lower percentage of dye coverage indicates improved protection from external aerosol droplets. (C) Relative exposure of the mannequin to aerosols assessed by the relative area covered by aerosolized fluorescent dye. Results represent the mean ± SD of 6 repeated trials. * and **** indicate adjusted P < 0.05 and P < 0.0001 compared via a one-way ANOVA with Tukey post-hoc test.

To assess the impact of improved mask fit on protection from external aerosols, the 3PM and UFM were challenged with fluorescent droplets (Fig 4). Exposure to aerosol droplets without any protection resulted in coverage of 77 ± 7% of the mannequin surface and 3PM masks reduced the dye-covered area by 22% (Fig 4B) (P < 0.05). Dye accumulation was observed in the same zones of poor fit identified during thermal imaging. This observation demonstrates that these areas of poor fit provide an opportunity for dangerous aerosols to bypass the mask filter material. The UFM was three times more effective at reducing aerosol ingress compared to the 3PM (Fig 4C) (P < 0.0001), even though the hard plastic of the mannequin reduced the ability of the UFM to form the tight seal normally generated as the mask is pulled against compliant skin by the adjustable tethering device.

### Quantitative fit test

**Fig 5:**
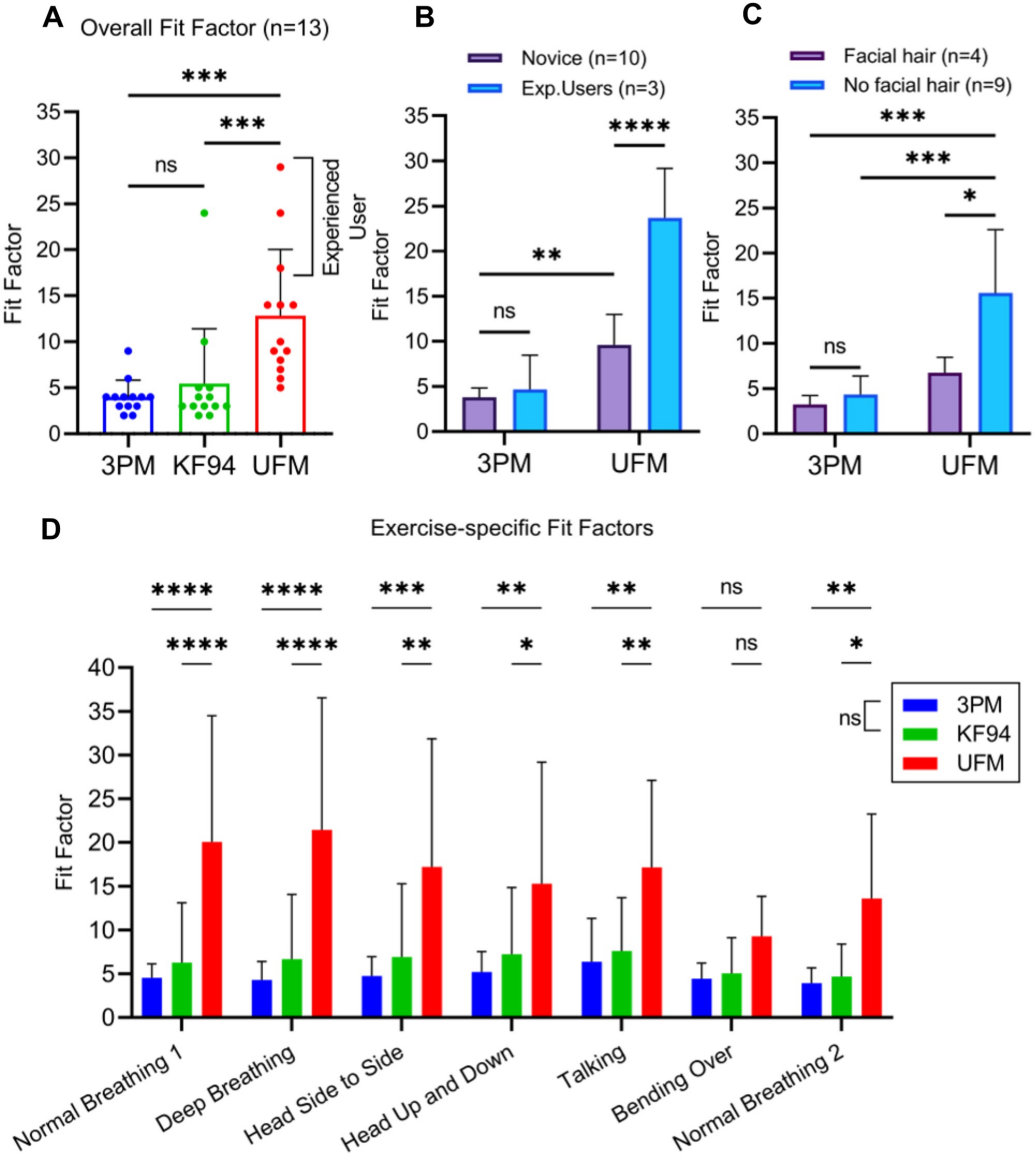
Quantitative fit test results of 3PM, KF94 and UFM. (A) Overall fit factors of quantitative fit test by PortaCount^®^ 8038 for n = 13 volunteers with 3PM, KF94 and UFM. (B) Fit factors between novice (n = 10) and experienced users (n = 3). (C) Effects of facial hair with 3PM and UFM groups. UFM with no hair outperformed both 3PM with and without hair and as well as UFM with hair. was used. (D) Exercise-specific fit factors measured with PortaCount^®^ 8038 of 3PM, KF94 and UFM. After reading a provided rainbow passage (‘Talking’), a non-measuring grimace exercise was performed per the OSHA fit test protocol. The post-grimace normal breathing exercise was labelled as ‘Normal Breathing 2’ to differentiate from the earlier normal breathing (‘Normal Breathing 1’). Bars are of the mean with error bars indicating the standard deviation. A Friedman test was performed for statistical analysis. *, **, ***, and **** indicate adjusted p-values of less than 0.05, 0.01, 0.001, and 0.0001, respectively.

We performed quantitative fit testing using a PortaCount^®^ fit tester. 13 volunteers underwent standard fit testing of 3PM, KF94 and UFM samples using the methods outlined in 29 CFR 1910.134, which is the standard minimal risk fit testing method used in non-research employment contexts. We considered this and the subsequent user surveys to be IRB exempt under categories 2 and 3 as defined in 45 CFR 46.101(b)(#). Subsequent IRB review of the testing protocol confirmed IRB exempt status. The average UFM fit factor (FF) was 12.9 ± 7.2 (Fig 5A), which was 320% of the 3PM FF (FF = 4.0 ± 1.8, P < 0.001) and 240% of the KF94 mask FF (FF = 5.5 ± 5.9, P < 0.001) respectively, while the observed difference between the 3PM and KF94 mask FFs was not statistically significant (P > 0.9999) (Fig 5A).

During the ‘Bending Over’ exercise, all three masks had a lower FF, and the UFM FF did not show a significant improvement compared to the other two masks (Fig 5D) (P > 0.05). Bending-over involved moving the upper body while a hose was connected to the mask during the test. Facial leakage was introduced due to the large body motion and strain created by the experimental setup in this position. Three experienced UFM users, whom as test users didn’t require donning instructions, scored an average overall FF of 23.7 ± 5.5 (P < 0.001) whereas novice UFM users scored an average overall FF of 4.7 ± 2.5, suggesting further face fit improvement with experience (Fig 5C). Interestingly, in novice users the UFM FF was 2.5 times higher than the 3PM FF (P < 0.005), consistent with a marked improvement in performance, even in novice users. While, in our user group, the presence of facial hair reduced fit factors, which was consistent with previous finding [23] (Fig 5D), the UFM FFs were still significantly higher compared to 3PM FFs in both with and without facial hair groups (P < 0.001 and P < 0.0001, respectively). Overall, the UFM group significantly outperformed the 3PM and the KF94 groups, regardless of familiarity, facial hair, or body movement.

### User evaluation

To assess ease of donning and perceived comfort, we conducted a survey of individuals that had previously used the UFM. Each user had thus viewed the donning instructions and had experience using the UFM (S3A Fig). Most responded that the UFM was easier or similar in difficulty to don compared to conventional 3PM, with only 13% reporting more difficulty donning the UFM (S3B Fig). More than 90% of the respondents found the UFM similar or superior in comfortability compared to the 3PM disposable mask (S3C Fig). With appropriate instruction provided, ease-of-use for UFM was comparable to the 3PM.

### Modeling UFM impact on community spread of an airborne pathogen

**Fig 6:**
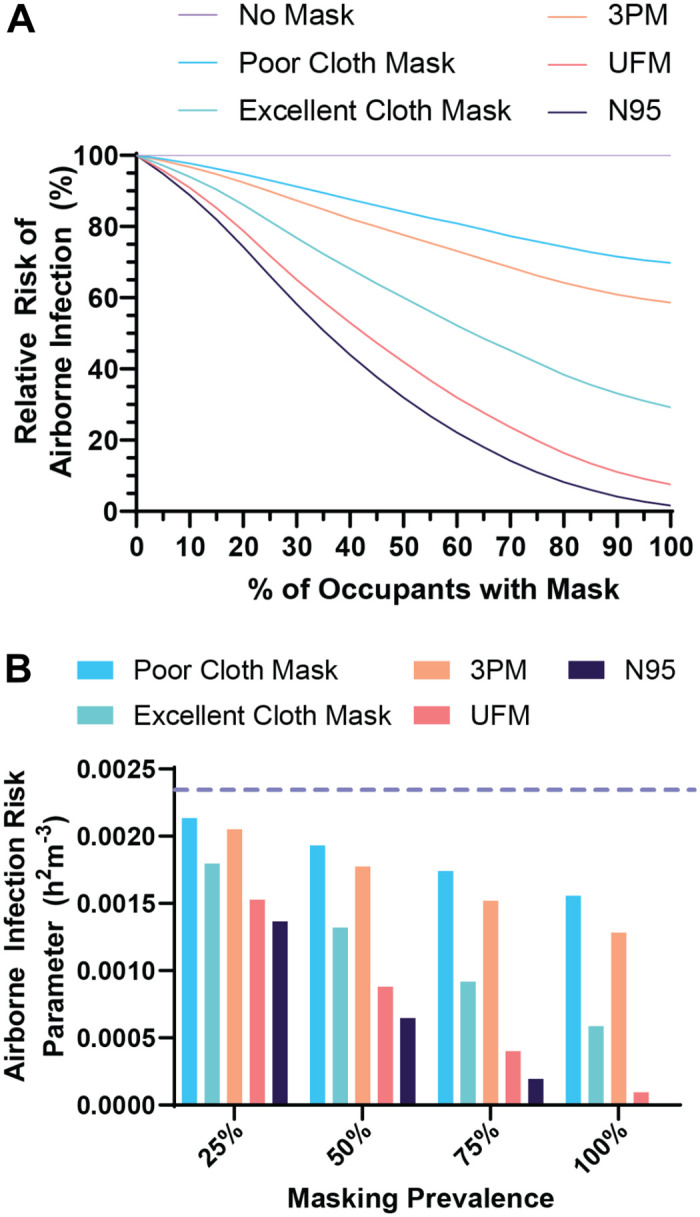
Model of airborne infection risk by mask type and usage. (A) The relative risk of a new airborne infection occurring in a classroom type environment with varying rates of masking and mask types. (B) The calculated individual infection risk parameter for individuals riding a subway train, assuming the presence of an infective individual. The dashed purple line indicates the risk parameter for no masking.

To evaluate the potential impact of the UFM on the risk of infection in the context of a classroom and mass transit, we used a model of aerosol disease transmission using parameters obtained from the current COVID-19 pandemic [19]. Masking was beneficial in the model no matter the quality of the mask, but as fit and filtration efficiency increased the amount of protection increased (Fig 6A). At with 50% of the class using masks the cloth mask, 3PM, excellent cloth mask, UFM, and N95 respirator reduced the relative risk of airborne infection by 18%, 24%, 44%, 62%, and 73% compared to no masks being used, respectively (Fig 6A). We calculated the relative impact of each mask type on the airborne infection risk parameter, a predictive measure of the rate an individual is exposed to an infectious agent, for a subway car. We found similarly that the UFM outperformed both model cloth masks and the 3PM (Fig 6B). At 50% mask prevalence the UFM use dropped the airborne infection risk by 50.4% compared to the 3PM. In both model situations, the UFM approached the efficacy of N95 respirators to prevent the community spread of COVID-19.

## Discussion

Poor mask fit has been a significant challenge for reducing the community spread of airborne pathogens. The most prevalent type of commercially available mask, disposable multi-layered face masks, such as 3PM, are plagued by poor fit issues [24] and while mask fit hacks such as double-masking or knotting tricks increase protection they are infrequently used [25]. To address these persistent design challenges, we have developed a low-cost high-performance mask suitable for mass production and widespread community adoption.

From a series of comprehensive performance evaluations, including leakage visualization and measured FFs, we have shown that the UFM tripled the protection from aerosols compared to standard disposable face masks, for only $0.05 in additional material cost per mask. This additional cost excludes costs of equipment, labor, and transportation. Each design element of the UFM was intentionally developed with a focus on manufacturability and scalability to enable the UFM to be produced on existing automated production lines and on standalone ultrasonic stations. As a result, large numbers of UFM can be produced at scale comparable to existing multi-layered face masks. The UFM is a low-cost, mass-producible, easy-to-don, and comfortable face mask that minimizes face seal leakage by (i) adding pliability to mask periphery to enable face molding to create a personalized facial fit, (ii) integrating ear loop tension adjustment facilitating optimal compression of the mask on the wearer’s face, and (iii) an inner "cough-trap” flap for blocking rapid respiratory jets induced by coughing or sneezing. With these three innovations, the performance of the UFM exceeds ASTM F3502 Level 2 Barrier Face Covering specifications and achieves the NIOSH criteria for a Workplace Performance Plus mask. While the quantitative fit experimental protocols implemented in the present study represent industry standards for evaluating face mask-fit performance, we performed additional experiments using laser particle scattering, thermal imaging, and fluorescent photography to visually illustrate the ability of the UFM to prevent the expulsion and ingress of aerosols.

While much attention was paid to mask type and filter material during the COVID-19 pandemic, the experimental data suggest that absent proper fit, mask performance can be severely compromised. A study of the 9 most common respirator masks used by healthcare workers in France found that for five of the models, 92% of the units had FFs of less than 25 [26]. In our study, all UFM users showed improved FF and experienced UFM users had an average overall FF of 23.7 ± 5.5, performance similar to that seen in widely used consumer grade respirator. In our study, UFM FF was superior to those of 3PM or KF94 masks, the performance of which was degraded by leakage, an observation consistent with previous studies [7, 27].

Throughout the COVID-19 pandemic, a wide range of alternative innovations designed to improve face mask fit have been developed. For example, face scanning technology was used to produce a user-customized mask brace, which is worn on top of the mask to minimize leakage [28]. Despite the advantages offered through this approach, this innovative technology requires 3D printing of custom mask braces by the users and is therefore challenging to deploy on a massive scale. Other notable innovations include the iMASC [29], a transparent sterilizable respirator, and a sew-free origami mask [30] that address face seal leakage issues by utilizing material’s natural properties. Elastic bands have also been developed to help seal masks but comfort and the need for additional aftermarket elements have limited their adoption [31]. While all of these examples are valuable innovations that improve individual protection, it is presently uncertain if these designs can be produced at the scale necessary for community respiratory protection which will require rapid deployment of thousands of units.

Mask design requires optimization across multiple performance domains, including filtration efficacy, breathing resistance, leakage, comfort, sustainability, cost, and supply chain robustness, with the added challenge of uniformly high performance across a wide range of face sizes and shapes. We assessed the capacity of our mask fit system (peripheral moldable elements, integrated tethering adjustment, and inner flap) to augment the performance of conventional 3PM, based on the knowledge that (a) conventional 3PM are widely available and (b) ASTM-F2100-compliant 3PM have been shown to have excellent filtration performance characteristics [32], implying that remediating face fit deficiencies in these masks would be most likely to produce a high-performing mask suitable for widespread community adoption. For the present study, quantitative fit testing was limited to the methods described in the OSHA fit test protocol for evaluating mask fit, and while these methods cover a wide range of scenarios that are reasonably representative of expected working activities, they do not include leisure activities such as jogging.

The tight fit of N95 masks creates discomfort and pressure injuries with extended and repeated use, a problem noted during the COVID-19 pandemic [9]. A survey of 4,306 clinicians in China during February 2020 found that 42.8% had experienced skin injuries due to their personal protective equipment [10]. Respiratory protection provided by the N95 respirators is dependent upon proper fit. Also, new users must repeatedly test different N95 respirator types to find a model that passes fit testing, after which each user may be required to go through an annual qualitative or quantitative fit test. If no fit test is performed, N95 respirator performance may be substantially reduced. Unfortunately, N95 respirators frequently need to be readjusted as 10–70% of initial tests fail to meet required levels depending upon the mask model and institution [26]. Moreover, N95 respirator access was limited during the pandemic, due to the needs of healthcare personnel operating in high-risk environments. These issues with N95 respirators, are part of the reason why even after shortages have somewhat resolved they still lack adoption in the broader community in low-risk settings and would be difficult to rapidly deploy for use by the general population. The value comparison of the UFM, the N95 respirator and alternative devices (S1 Table) shows that the UFM may address many of these challenges. In models of community spread, the UFM approached the same level of protection as N95 masks to prevent the spread of airborne disease on a community basis. However, in an individual context, a fit-tested N95 still offers superior protection for use in aerosol-generating environments [9].

We used multiple methods to illustrate improved fit and respiratory protection with the UFM. While we developed the formable elements with the intent of achieving as close to a universal fit as possible, further validation is still needed. We were limited in the variety of face shapes and by the number of recruitable volunteers during the pandemic. Also, assessing the effectiveness of cough-trapping flap and its design was limited by a lack of a means to generate consistent coughs. Our modeling of individuals posed by the masks is limited by many of the assumptions specified in our methods that were necessary to facilitate calculation. Additionally, modeling assumed that all COVID-19 infection occurs via aerosol transmission neglecting the risk of deposition on mucous membranes and contact transmission from the hands, which contribute to the spread of COVID-19 [33]. In both the subway and classroom scenarios, there was assumed to be exactly one contagious individual present, constant uniform spacing among individuals, uniform distribution of infectious particles within the space, and proper mask utilization. However, these considerations do not accurately match reality, where there may not be no infectious or potentially multiple infectious individuals present.

## Conclusion

To the best of our knowledge, this work represents the first mass-producible barrier face covering achieving conformity to the NIOSH Workplace Performance Plus criteria and the ASTM F3502 Level 2 enabled by three design elements in a highly scalable face mask design: (i) a peripheral moldable element, (ii) an integrated ear loop tension adjustment, and (iii) an inner ‘cough-trapping’ flap layer. In combination with current face mask materials, the UFM design features significantly improved mask performance without compromising comfort at low cost by reducing both the emission and ingress of particles and aerosols. In the context of community protection from the spread of airborne disease, the UFM approached the protection provided by the N95 respirator.

## Supporting information

S1 Fig Different donning styles with the Ultra fit Mask for improved fitting. (A) C-shaped: C-shaped curve on the side edges with pinching the chin edge. (B) V-shaped: pinching the side wires resembling a V. Optional chin pinching may further tighten fitting depending on the user’s preferences. (C) L-shaped: folding the lower parts of the side wires resembling an L. Optional chin pinching may further tighten fitting depending on user’s preferences.
(TIF)

S2 Fig Three different face masks (3PM, KF94, UFM) used for quantitative fit test donned on an ISO 16976–2 medium headform. (A) 3PM. Large gaps on the cheek sides are clearly visible. (B) KF94. Side gaps are visible. Infraorbital-nasal region and the chin appear closed. (C) UFM. The mask showed reduced side gaps and was tucked under the chin by pinched chin wire.
(TIF)

S3 Fig User validation survey with 15 participants. (A) A majority of respondents (72%) used a conventional 3PM. (B, C) >50% of respondents replied ‘equal or easier to don’ an UFM and ‘equal or more comfortable’ to wear an UFM compared to the 3PM.
(TIF)

S1 Table Value comparison. (DOCX)

S1 Movie A movie of 3-ply mask and Ultra fit mask on a rotating turntable. (MP4)

S2 Movie A movie of exhaled vapor tracking of the front and side views. (MP4)

S3 Movie Infrared thermal imaging movie with 3-ply mask and Ultra fit mask. (MP4)
