# Supplementary figures and images for: The Ultra fit community mask—Toward maximal respiratory protection via personalized face fit
Source: PLoS One. 2023 Mar 15;18(3):e0281050. doi: 10.1371/journal.pone.0281050 (PMC10016631; doi:10.1371/journal.pone.0281050)

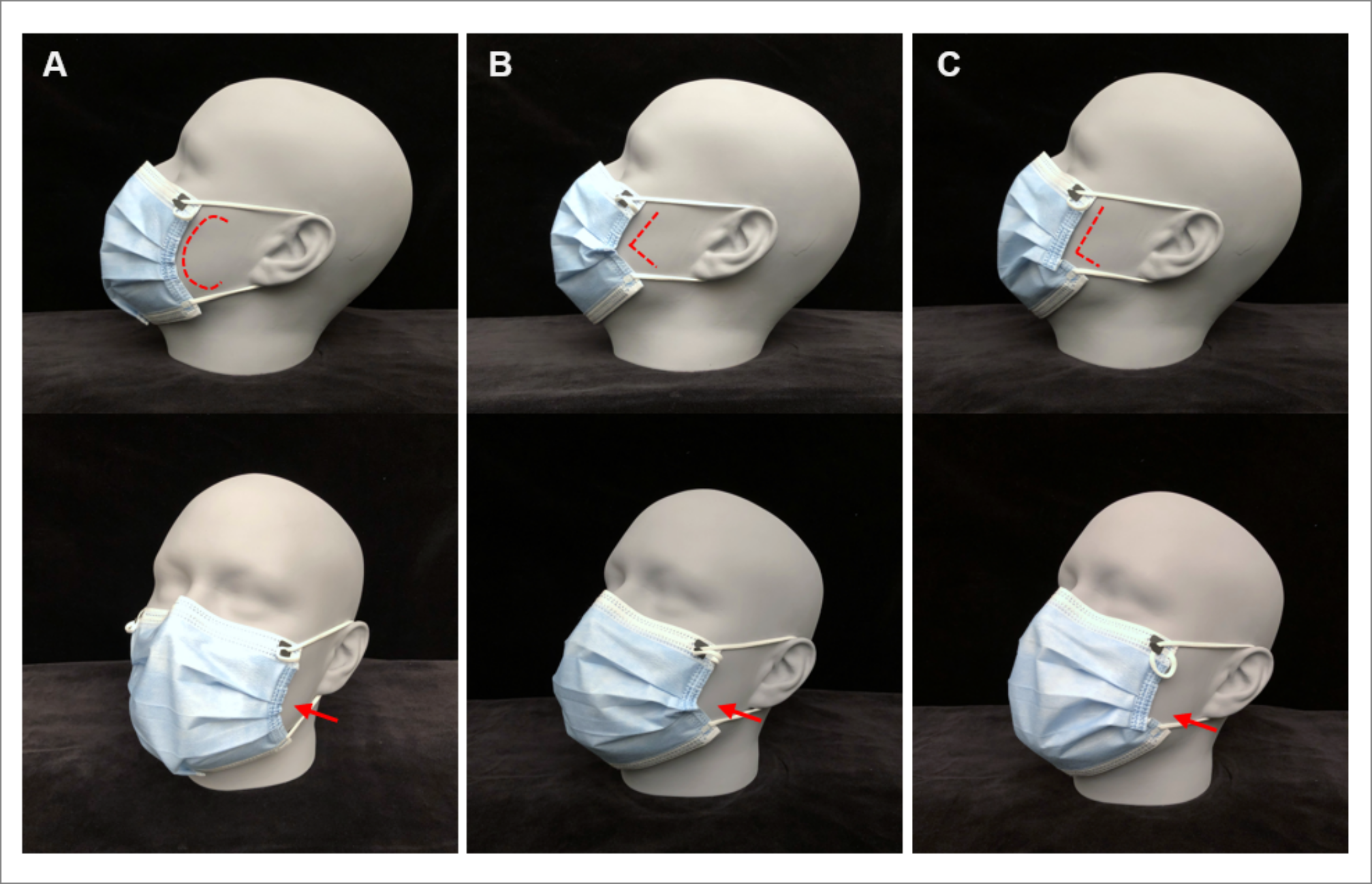

Supplement: S1 Fig — (A) C-shaped: C-shaped curve on the side edges with pinching the chin edge. (B) V-shaped: pinching the side wires resembling a V. Optional chin pinching may further tighten fitting depending on the user’s preferences. (C) L-shaped: folding the lower parts of the side wires resembling an L. Optional chin pinching may further tighten fitting depending on user’s preferences. (TIF) [file pone.0281050.s001.tif]

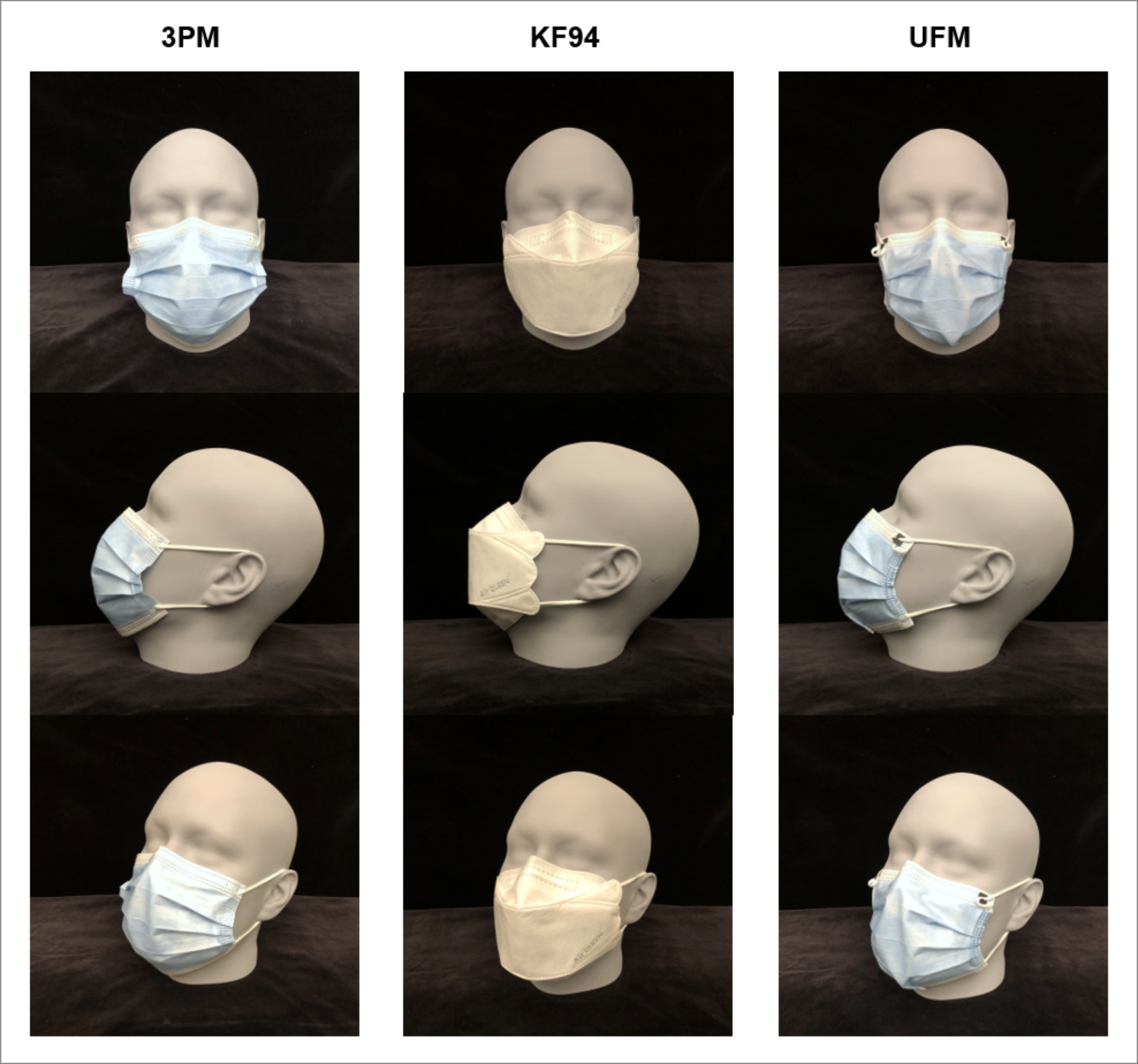

Supplement: S2 Fig — (A) 3PM. Large gaps on the cheek sides are clearly visible. (B) KF94. Side gaps are visible. Infraorbital-nasal region and the chin appear closed. (C) UFM. The mask showed reduced side gaps and was tucked under the chin by pinched chin wire. (TIF) [file pone.0281050.s002.tif]

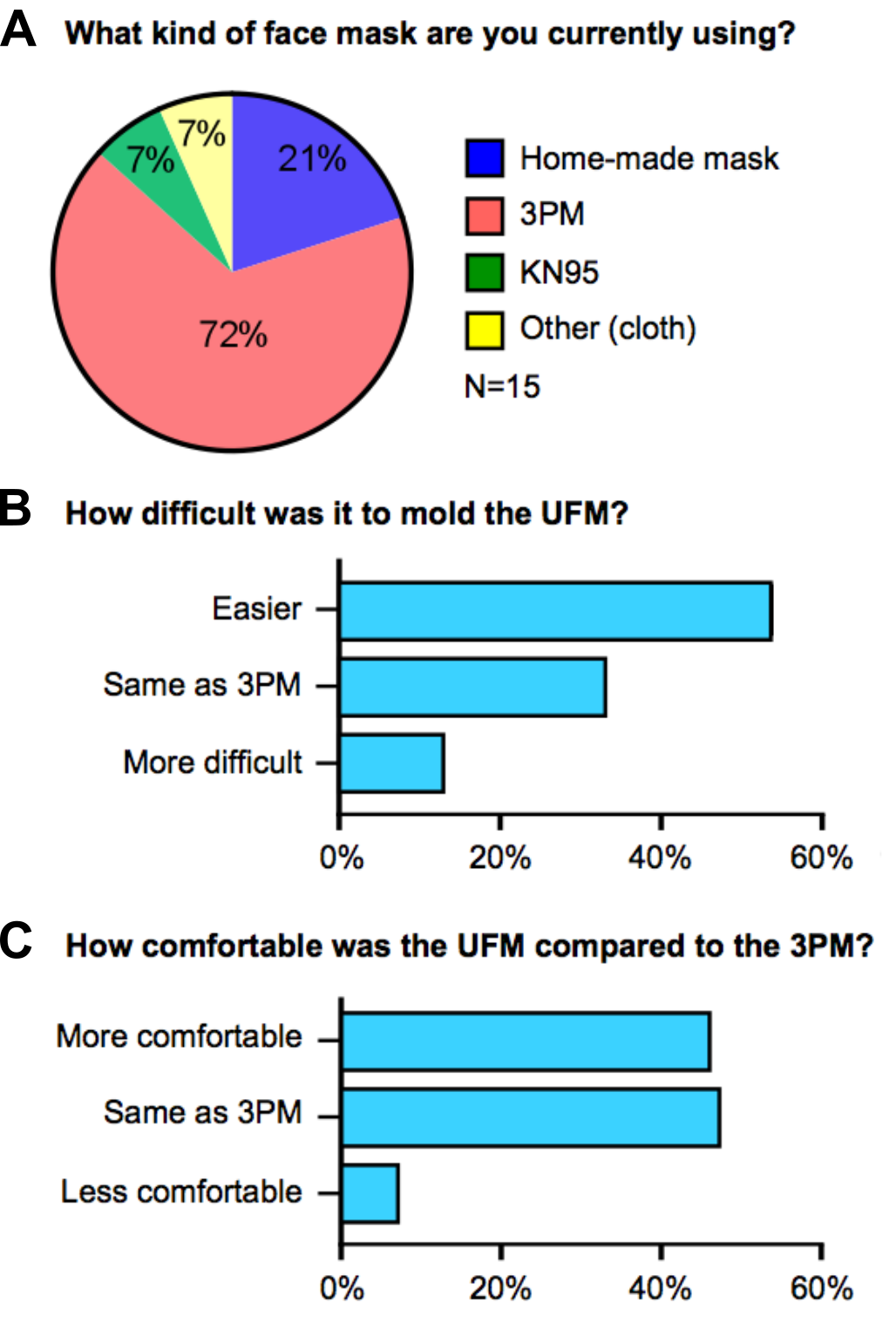

Supplement: S3 Fig — (A) A majority of respondents (72%) used a conventional 3PM. (B, C) >50% of respondents replied ‘equal or easier to don’ an UFM and ‘equal or more comfortable’ to wear an UFM compared to the 3PM. (TIF) [file pone.0281050.s003.tif]
