# Supplementary material for: The Ultra fit community mask—Toward maximal respiratory protection via personalized face fit
Source: PLoS One. 2023 Mar 15;18(3):e0281050. doi: 10.1371/journal.pone.0281050 (PMC10016631; doi:10.1371/journal.pone.0281050)
Supplement: S1 Table — (DOCX) [file pone.0281050.s004.docx]

**S1 Table.** Value Comparison

|  | **Ultra Fit Mask** | **Conventional Mask**  (e.g. cloth masks, 3-ply masks, KF94) | **Clinical Grade Respirators**  (e.g. N95, KN95, FFP3) | **Alternative Solutions**  (e.g. mask fitter, double-masking, knotting) |
| --- | --- | --- | --- | --- |
| **Total Inward Leakage (excluding filter penetration leakage)** | 10.0% ± 4.9% | 20-40% [1,2] | < 1% (if fit test passed)  If fit test failed, 4% or higher [3]  Initial fit test pass rate: 83% [4] | Can achieve < 1% in conjunction with quality disposable mask [5] |
| **Filtration** | 95.98% (at 85 lpm per ASTM F3502**^a^**) | 25-80% [6], specified at 95%**^b^** | > 95% (at 85 lpm)  NIOSH test method**^a^** | N/A |
| **Fit (Fit Factor, FF)** | 12.9 (n=13) | 2.6 - 4.4 [1,2] | >100 (if passed a fit test)  < 25 (if failed a fit test) [3] | Help achieving mask FF >100 [5] |
| **Breathing Resistance** | 5.8 mmH_2_O (at 85 lpm per ASTM F3502**^a^**) | varies (4-27 mmH_2_O)**^c^** | <35 mm H2O column (at 85 lpm) | High/Medium |
| **Comfort** | Good | Good | Poor | Medium or Poor |
| **Accessibility to public** | Good | Good | Poor | Good |
| **Manufacturing at scale** | Good | Good/Poor | Intermediate | Good or N/A |
| **Cost** | Low (~$0.50, $0.05 addition to base mask price) | High for cloth mask (>$0.50)  Medium for KF94 (~$0.50)  Low for 3-ply mask (<$0.50) | High (>$1.00)  Fit testing cost: $30-$50 per person annually [4]. | High (>$1.00) |
| **Easiness-to-use** | Good | Good | Intermediate | Intermediate |
| **Washability/**  **Resuability** | Yes | Cloth: Yes  Non-cloth: No | Yes [7] | Depends |
| **Contact Dermatitis Risk** | No | No | Yes [8,9] | No |
| **Applicable Standards/Certification** | ASTM F3502 Level 2 for both filtration & breathability.  NIOSH Workplace Performance Plus mask | Foreign standards e.g. GB/T 32610- 2016, YY/T 0969-2013, GB2626-2006, KF94, AFNOR SPEC S76-001 | Surgical N95 designation, N95 require fit testing | N/A |

^a^ ASTM F3502-21, “Standard Specification for Barrier Face Coverings”

^b^ Bacterial filtration efficiency per ASTM F2101 or equivalent foreign standards

^c^ observed by the authors using TSI 8130 and NIOSH TEB-APR-STP-0059.

# SI References

1. Lawrence RB, Duling MG, Calvert CA, Coffey CC. Comparison of performance of three different types of respiratory protection devices. J Occup Environ Hyg. 2006;3(9):465–74.

2. Oberg T, Brosseau LM. Surgical mask filter and fit performance. Am J Infect Control. 2008;36(4):276–82.

3. Ciotti C, Pellissier G, Rabaud C, Lucet JC, Abiteboul D, Bouvet E. Effectiveness of respirator masks for healthcare workers, in France. Med Mal Infect [Internet]. 2012;42(6):264–9. Available from: http://dx.doi.org/10.1016/j.medmal.2012.05.001

4. Wilkinson IJ, Pisaniello D, Ahmad J, Edwards S. Evaluation of a Large-Scale Quantitative Respirator-Fit Testing Program for Healthcare Workers: Survey Results. Infect Control Hosp Epidemiol. 2010;31(9):918–25.

5. Fix The Mask Data —the Essential Mask Brace | by Fix The Mask — the technical. | Medium [Internet]. [cited 2021 Mar 17]. Available from: https://fixthemask.medium.com/fix-the-mask-data-the-essential-mask-brace-ad5bdf33f506

6. Clapp PW, Sickbert-Bennett EE, Samet JM, Berntsen J, Zeman KL, Anderson DJ, et al. Evaluation of cloth masks and modified procedure masks as personal protective equipment for the public during the COVID-19 pandemic.

7. N95DECON. N95 Decontamination & Reuse Method Decision Matrix. 2020.

8. Yu J, Chen JK, Mowad CM, Reeder M, Hylwa S, Chisolm S, et al. Occupational dermatitis to facial personal protective equipment in health care workers: A systematic review. J Am Acad Dermatol [Internet]. 2021;84(2):486–94. Available from: https://doi.org/10.1016/j.jaad.2020.09.074

9. Hornbeck A, Cichowicz JK, Kilinc-Balci S, Rottach D, Pollard J, Boyles H. Skin Irritation from Prolonged Use of Tight-Fitting Respirators | Blogs | CDC [Internet]. 2020 [cited 2021 Jun 21]. Available from: https://blogs.cdc.gov/niosh-science-blog/2020/08/04/skin-irritation-respirators/
